# Palliative care in the intensive care unit: fundamental aspects, ethical frameworks, and challenges in the Brazilian context

**DOI:** 10.3389/fmed.2026.1914706

**Published:** 2026-09-01

**Authors:** Brenno Cardoso Gomes, Anibal Basile Filho, Joao M. Silva

**Affiliations:** 1 Universidade Federal do Paraná, Departamento de Clínica Médica, Curitiba, Brazil; 2 Universidade de São Paulo, Campus de Ribeirão Preto, Ribeirão Preto, Curitiba, Brazil; 3 Universidade de São Paulo, São Paulo, Brazil

**Keywords:** Brazil, intensive care, palliative care, terminal care, withdrawal of treatment, withholding treatment

## Abstract

Significant medical advancements over the last century have reduced in-hospital mortality but raised concerns about prolonged hospitalizations and aggressive treatments potentially compromising the quality of life of critically ill patients. In Brazil, healthcare professionals often encounter ethical dilemmas and hesitancy when navigating orthothanasia and life-sustaining treatment decisions. This review examines the fundamental aspects of palliative care in the Intensive Care Unit (ICU), addressing the research question: *What are the fundamental aspects of palliative care in the ICU?* The study is positioned as a narrative review with systematic evidence collection, allowing for a multidimensional synthesis of clinical evidence, ethical frameworks, and regulatory challenges. The review focuses on four interrelated dimensions: (1) core clinical components (symptom management, communication, and decision-making); (2) ethical frameworks, specifically principalism and virtue ethics, guiding end-of-life decisions; (3) spiritual and existential care; and (4) contextual challenges in the Brazilian ICU setting, including barriers to life support limitation and strategies to prevent dysthanasia. Data were gathered from systematic searches in PubMed (MEDLINE), Scopus, LILACS, and the Virtual Health Library (VHL), spanning publications from 2004 to 2024, supplemented by legal resolutions and clinical guidelines. From 111 articles identified after deduplication, 36 were selected for full-text analysis following independent screening by two researchers. Key findings underscore the importance of structured communication, individualized symptom management protocols, and the role of virtue-based competencies in mitigating moral distress. Context-specific barriers in Brazil include legal uncertainty and training deficits. The reviewed evidence supports the implementation of structured goals-of-care conferences and interdisciplinary protocols to improve care quality and prevent dysthanasia.

## Introduction

In the last century, technological advances capable of sustaining life despite organ failure have drastically reduced hospital mortality. However, these advances have also generated important discussions about the impacts of prolonged hospitalizations and aggressive treatments in Intensive Care Units (ICUs) on patients’ quality of life. There is growing concern about the detrimental effects of adopting measures considered futile that can artificially prolong life at the expense of the physical and psychological suffering of patients and the emotional suffering of family members and caregivers (1, 2).

Palliative care represents a patient-centered, rather than disease-centered, model that is essential for the management of progressive and incurable diseases, with a primary focus on optimizing quality of life. According to the World Health Organization (WHO), palliative care aims to alleviate suffering — whether physical, psychological, spiritual, or social — and its comprehensive implementation should be a global health priority. WHO emphasizes that palliative care may be necessary for any cause of suffering and should be accessible at all levels of health (3).

In the tertiary health sector, palliative care in the ICU, while necessarily included in the context of critical illness and the dying process, should not be used as a synonym for end-of-life care or limitation of advanced life support alone (4). Palliative care in the ICU encompasses a broader set of interventions delivered by interdisciplinary teams to alleviate suffering, clarify care goals, and support families while critical care therapies are continued, limited, or withdrawn. Core components include daily symptom assessment and management, structured communication about prognosis and treatment options, shared decision-making processes, family support programs, and staff education (5, 6).

When advanced life support does not achieve cure or reduce the duration and consequences of hospitalization in the ICU, the dignity of patients and their caregivers can be deeply compromised. In this context, the focus of treatment should shift to optimizing comfort measures, respecting the limits and inherent fragility of the patient, as well as their natural dying process. This change in treatment priorities reflects the central philosophy defended by palliative medicine, which, in certain cases, can culminate in the decision to withdraw life support or choose not to institute it in the face of clinical worsening (7).

Despite the international consensus on the ethical appropriateness of withholding and withdrawing life-sustaining treatments when they no longer meet the patient’s goals, significant barriers to translating these principles into practice persist, especially in the Brazilian context. Brazilian ICUs face particular obstacles, including legal uncertainty, inadequate training in end-of-life care, staff fragmentation, low utilization of do-not-resuscitate (DNR) orders, and cultural reluctance to withdraw versus withhold treatments (8–10). These barriers contribute to unresolved ethical tensions around decision-making authority, perceived futility, and moral distress among health professionals (11, 12).

In addition, the traditional bioethical frameworks based on the four principles — autonomy, beneficence, nonmaleficence, and justice — although necessary, may prove insufficient to guide clinicians in the complex and emotionally charged situations characteristic of end-of-life care in the ICU. Virtue ethics offers a complementary approach by emphasizing clinicians’ character, practical wisdom, and the goal of human flourishing, helping to navigate situations where principles conflict or provide inadequate guidance (13, 14). Similarly, spiritual care — which addresses existential suffering, meaning-making, and religious needs — is increasingly recognized as an integral part of comprehensive palliative care, but remains underutilized in many ICU settings (15, 16).

A fundamental challenge for both clinical practice and scholarly discussion in this field is the heterogeneity of terminology across traditions. In the Anglo-American tradition, the central concepts are withholding life-sustaining treatment — the deliberate decision not to initiate an intervention that could prolong life, because its burdens are deemed to outweigh its benefits in light of the patient’s goals — and withdrawing life-sustaining treatment — the decision to discontinue an already-initiated life-sustaining intervention under the same proportionality rationale. Brazilian regulatory and ethical discourse is organized primarily around the concept of orthothanasia, defined as the allowance of natural death without the employment of disproportionate or extraordinary interventions, legally and ethically distinct from euthanasia (which involves active hastening of death) and from negligent omission of care. The opposite pole — the prolongation of the dying process through disproportionate, burdensome, and futile interventions, resulting in suffering without meaningful benefit — is captured by the Brazilian concept of dysthanasia. The Spanish-language Latin American tradition has largely adopted the term *limitación del esfuerzo terapéutico* (limitation of therapeutic effort) or *adecuación del esfuerzo terapéutico* (adequation of therapeutic effort), shifting the emphasis toward collaborative clinical deliberation. These terminological differences carry implications for literature retrieval, clinical communication, and comparative research, and were intentionally addressed in the search strategy of the present review by incorporating tradition-specific descriptors across all three languages.

This narrative review addresses the research question: *What are the fundamental aspects of palliative care in the ICU?* The review examines four interrelated dimensions: (1) core clinical components (symptom management, communication, decision-making); (2) ethical frameworks (virtue ethics and principalism) guiding end-of-life care; (3) spiritual and existential care; and (4) contextual barriers and evidence-based strategies specific to the Brazilian ICU setting, including life support limitation and the prevention of dysthanasia.

## Methods

### Study design and rationale

This study used a narrative review methodology to comprehensively examine the fundamental aspects of palliative care in the ICU. A narrative review approach was chosen for its capacity to integrate diverse types of evidence — including empirical studies, ethical analyses, clinical guidelines, legal frameworks, and theoretical perspectives — necessary to address the multifaceted nature of palliative care in the ICU (17, 18).

While this article incorporates systematic review procedures—such as structured database searches, predefined eligibility criteria, independent screening by two reviewers, and a PRISMA-compatible flow diagram—to ensure rigor and minimize selection bias, it is methodologically positioned as a narrative review with systematic evidence collection.

This hybrid design is justified by the review’s objective: to provide a broad, multidimensional synthesis of clinical, ethical, and regulatory aspects (19, 20). Such conceptual frameworks (e.g., virtue ethics, cultural barriers) involve multifaceted interpretations that are not suitable for the rigid quantitative synthesis or the narrow focus typical of a standard systematic review. This narrative review was conducted with attention to transparency and reproducibility, incorporating elements of the SANRA framework (Scale for the Assessment of Narrative Review Articles) (17). The methods section provides explicit details about searched databases, search terms, date ranges, inclusion/exclusion criteria, screening process, number of identified and selected articles, and synthesis approach to allow readers to judge the comprehensiveness and potential biases of the literature review.

### Research question

The review was guided by the explicit research question: *“What are the fundamental aspects of palliative care in the ICU?”* This question was operationalized to encompass: (1) core clinical components (symptom management, communication, decision-making); (2) ethical frameworks (virtue ethics and principalism) guiding end-of-life practice; (3) dimensions of spiritual and existential care; and (4) contextual challenges and evidence-based strategies, with special attention to the Brazilian scenario, including life support limitation and prevention of dysthanasia.

### Literature search strategy

#### Databases searched

Systematic searches were carried out in four electronic databases: PubMed (MEDLINE), Scopus, LILACS (Health Sciences Literature of Latin America and the Caribbean), and the VHL (Virtual Health Library).

#### Search terms

Search strategies combined terms related to palliative care, intensive care, and end-of-life decision-making across three languages. The complete, structured search term list is presented in Table 1. The terms were combined using Boolean operators (AND, OR) and adapted to the indexing system of each database and to the controlled vocabulary (e.g., MeSH terms in PubMed; DeCS — *Descritores em Ciências da Saúde* — in LILACS and VHL).

**Table 1:** Structured search terms used across databases (English, Portuguese, and Spanish).

Domain	English	Portuguese	Spanish
Palliative care	Palliative care	Cuidados paliativos	Cuidados paliativos
Intensive care	Intensive care unit; ICU; critical care	Unidade de terapia intensiva; UTI; cuidados intensivos	Unidad de cuidados intensivos; UCI; cuidados críticos
Terminality/End of life	Terminality; end-of-life care; terminal care	Terminalidade; cuidados de fim de vida	Terminalidad; cuidados al final de la vida
Treatment limitation	Treatment withholding; withdrawal of treatment; life support limitation	Suspensão de tratamento; limitação de suporte de vida; retirada de tratamento	Limitación del esfuerzo terapéutico; adecuación del esfuerzo terapéutico; suspensión del tratamiento
Decision concepts	Do-not-resuscitate (DNR); goals of care; medical futility	Ordem de não ressuscitar; objetivos do cuidado; futilidade médica	Orden de no reanimación; objetivos del cuidado; futilidad médica
Brazilian/ethical concepts	Orthothanasia; dysthanasia	Ortotanásia; distanásia	Ortotanasia; distanasia
Ethics	Virtue ethics; spiritual care; compassion	ética das virtudes; cuidado espiritual; compaixão	ética de la virtud; cuidado espiritual; compasión
Geographic	Brazil; Brazilian	Brasil; brasileira	Brasil; brasileño

#### Representative Boolean search string (PubMed/MEDLINE)

The following representative Boolean search string illustrates the search logic applied in PubMed (MEDLINE): (“Palliative care”[MeSH Terms] OR “palliative care”[Title/Abstract]) AND (“intensive care units”[MeSH Terms] OR “intensive care unit”[Title/Abstract] OR “ICU”[Title/Abstract] OR “critical care”[Title/Abstract]) AND (“end-of-life care”[Title/Abstract] OR “withholding treatment”[Title/Abstract] OR “withdrawal of treatment”[Title/Abstract] OR “life support limitation”[Title/Abstract] OR “do-not-resuscitate”[Title/Abstract] OR “orthothanasia”[Title/Abstract] OR “dysthanasia”[Title/Abstract] OR “goals of care”[Title/Abstract]) AND (“Brazil”[Title/Abstract] OR “Brazilian”[Title/Abstract]).

Analogous strings were adapted for Scopus (using TITLE-ABS-KEY fields), LILACS, and VHL (using DeCS controlled vocabulary), with appropriate Boolean operators and controlled vocabulary adjustments for each database’s indexing conventions.

#### Date range

The search spanned publications from January 2004 to December 2024, capturing two decades of evolving practice and ethical discourse in ICU palliative care.

#### Supplemental sources

In addition to database searches, the review incorporated:

- Legal documents and resolutions of the Federal Council of Medicine of Brazil (*Conselho Federal de Medicina*).
- Clinical practice guidelines of professional societies.
- Relevant content from palliative care and critical care textbooks.

### Inclusion and exclusion criteria

#### Inclusion criteria

- Studies addressing palliative care, end-of-life decision-making, or limiting life support in adult or pediatric ICUs.
- Empirical research (quantitative, qualitative, mixed methods).
- Ethical reviews, position papers, and guidelines relevant to palliative care in the ICU.
- Studies that examine barriers, facilitators, or cultural factors that influence palliative care in the ICU.
- Publications in English, Portuguese, or Spanish.
- Studies that address virtue ethics, compassion, or spiritual care in critical or palliative care settings.

#### Exclusion criteria

- Studies focused exclusively on settings outside the ICU with no relevance to critical care.
- Publications not available in full text.
- Studies with insufficient methodological detail or uncertain relevance to the research question.

#### Screening and selection process

The screening and selection process followed these steps:

1. *Database search*: Searches of the four databases yielded 111 articles after duplicates were removed.
2. *Screening of titles and abstracts*: Two researchers analyzed titles and abstracts independently in relation to inclusion and exclusion criteria. Disagreements were resolved through discussion.
3. *Full-text review*: Articles that passed the initial screening underwent full review by both researchers to assess eligibility and relevance to the research question.
4. *Final selection*: 36 articles were selected for detailed analysis and inclusion in the qualitative synthesis.
5. *Supplemental materials*: Additional legal documents, guidelines, and textbook chapters were included as needed to provide context and support specific claims.

## Results

### Literature search outcomes

**Figure 1:**
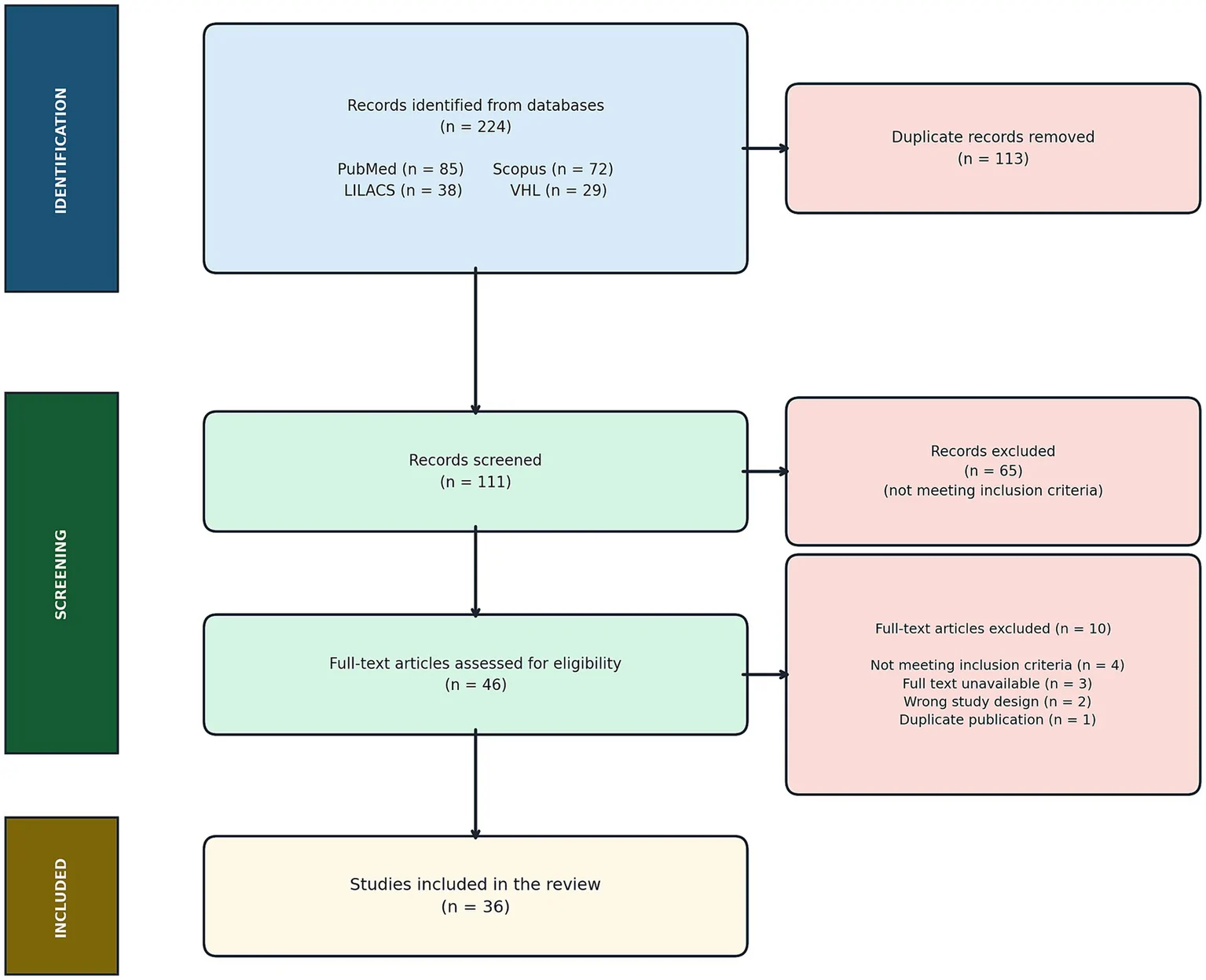
PRISMA-compatible flow diagram of article selection.

The search was conducted across four electronic databases — PubMed (MEDLINE), Scopus, LILACS, and VHL — covering publications from January 2004 to December 2024. The database-specific retrieval counts, after application of the search strategies described in the Methods section, are presented in Figure 1.

Following deduplication, 111 unique records were identified. These were screened independently by two researchers based on title and abstract; records that did not meet the inclusion criteria at this stage were excluded. Articles that passed the initial screening underwent full-text review for eligibility. A total of 36 articles were included in the final qualitative synthesis. The remaining articles were excluded after full-text review due to the following reasons: studies conducted exclusively in non-ICU settings; studies with insufficient methodological detail; publications not meeting the language inclusion criteria (English, Portuguese, or Spanish); and studies with uncertain relevance to the research question.

The full article selection process is illustrated in Figure 1 (PRISMA-compatible flow diagram).

### Characteristics of included studies

The 36 included studies were methodologically heterogeneous, comprising empirical studies (quantitative surveys, qualitative studies, and observational research), systematic reviews, ethical analyses, position papers, clinical guidelines, and legal documents. Studies addressed topics including: symptom management protocols; structured communication and family-centered care; ethical frameworks (including virtue ethics and principalism); spiritual care in the ICU; barriers and facilitators to palliative care implementation; the Brazilian legal and regulatory context; and comparative international data on end-of-life practices. Table 2 presents the key characteristics of the included studies.

**Table 2:** Characteristics of included studies (*n* = 36).

**#**	First Author (Year)	Country	Study design	Main topic	Key findings
1	Piva et al. (1)	Brazil	Multicenter observational	End-of-life practices in Brazilian ICUs	Life support limitation performed in minority of cases; legal and cultural barriers identified
2	Moritz et al. (2)	Brazil	Cross-sectional survey	Terminality and palliative care in the ICU	Insufficient palliative care education; low DNR use; discomfort with withdrawing treatment
3	Moritz et al. (8)	Brazil	Survey/attitudes study	Physicians’ attitudes toward end-of-life decisions	Fear of legal repercussions; preference for withholding over withdrawing
4	Lago et al. (9)	Brazil	Multicenter prospective	End-of-life in Brazilian pediatric ICUs	Lower life support limitation rates than international standards; cultural resistance documented
5	Moritz et al. (10)	Brazil	Forum/consensus report	End-of-life research group — 1st Forum	Identified team fragmentation and training gaps as primary barriers in Brazilian ICUs
6	Truog et al. (4)	United States	Consensus statement	ACCM recommendations for end-of-life ICU care	Established framework for palliative care integration in critical care; ethical equivalence of withholding/withdrawing
7	Aslakson et al. (6)	United States	Systematic review	Evidence-based palliative care interventions in ICU	Structured family meetings, communication training, and symptom protocols improve outcomes
8	Nelson et al. (5)	United States	Consensus/IPAL-ICU	Triage criteria for ICU palliative consultation	Early palliative care triggers improve quality of dying and family satisfaction
9	Gardiner (13)	United Kingdom	Ethical analysis	Virtue ethics in medical dilemmas	Virtue ethics complements principalism; practical wisdom guides complex decisions
10	Wilkinson and Savulescu (21)	UK/Australia	Ethical analysis	Withdrawing vs. withholding treatment	No ethical distinction between withholding and withdrawing; both justified by proportionality
11	Sprung et al. (29)	Europe	Multicenter survey	Attitudes toward end-of-life — ETHICATT study	Cultural variation in end-of-life attitudes across European countries and professional roles
12	Sprung et al. (57)	Europe	Multicenter observational	End-of-life practices in European ICUs — ETHICUS	Higher rates of treatment limitation in northern than southern Europe; distinct practice patterns
13	Balboni et al. (15)	United States	Mixed methods	Spiritual care assessment in palliative care	Spiritual distress prevalent; routine spiritual screening underused in ICUs
14	Sinclair et al. (39)	Canada	Grounded theory	Healthcare professionals’ experiences of compassion	Compassion model developed; relational, cognitive, and behavioral dimensions identified
15	Sinclair et al. (40)	Canada	Systematic review	Compassion training in health professions	Structured training measurably increases compassion competence; heterogeneity of evidence noted
16	Salman et al. (51)	Brazil	Narrative review	National Palliative Care Policy challenges	Significant gap between policy framework and implementation; training deficits identified
17	Lago et al. (58)	Brazil	Comparative review	End-of-life care in children: Brazil and international	Rates of life support limitation increasing but still below international standards
18	White et al. (59)	United States	Qualitative/observational	Shared decision-making in ICUs	Low family involvement in decisions; structured processes recommended to improve outcomes
19	Gutierrez (11)	United States	Qualitative	Moral distress in critical care nurses	Moral distress strongly linked to end-of-life decision conflicts; communication training recommended
20	Curtis and White (23)	United States	Practical guidance	Evidence-based family conferences in ICU	Structured family meetings improve understanding, reduce uncertainty, and support decision-making
21	Carlet et al. (7)	Europe/International	Consensus conference statement	Challenges in end-of-life care in the ICU	Identified key ethical and clinical challenges; endorsed palliative care integration alongside critical care; called for standardized end-of-life protocols
22	Puchalski et al. (16)	USA/International	Expert consensus/Guideline	Spiritual dimension of whole-person care	Proposed national/international consensus for integrating spiritual care into palliative practice; recommended routine spiritual screening and chaplaincy referral
23	Devlin et al. (24)	USA/International	Clinical practice guideline	Pain, agitation, delirium, immobility, and sleep (PADIS) in ICU	Evidence-based recommendations for PADIS management in adult ICU patients; emphasized non-pharmacological interventions and individualized assessment
24	Cherny and Radbruch (27)	Europe	Expert consensus/Guideline (EAPC)	Palliative sedation framework	Defined proportional palliative sedation standards; distinguished from euthanasia; established ethical safeguards and monitoring requirements
25	Bosslet et al. (22)	USA/International	Multiprofessional policy statement	Potentially inappropriate treatments in ICUs	Provided stepwise framework for responding to requests for inappropriate treatments; recommended communication-based approaches before unilateral clinical decision
26	Kopar et al. (62)	United States	Practical guidance/Review	Medical futility in the ICU	Proposed stepwise practical approach to futility discussions; emphasized institutional processes and multidisciplinary communication before unilateral decisions
27	Metaxas (63)	International	Narrative review	End-of-life problems in the ICU	Reviewed major end-of-life challenges; emphasized need for structured protocols, team communication, and formal palliative care integration in critical care settings
28	Blinderman and Billings (70)	United States	Clinical review	Comfort care for dying patients in the hospital	Outlined practical approach to comfort care; addressed symptom management, ethical considerations, and communication strategies for imminently dying patients
29	Scheunemann et al. (71)	United States	Systematic review	Communication interventions in the ICU	Identified effective components of structured family meetings; documented modest but consistent evidence for communication interventions improving family outcomes
30	Curtis et al. (72)	United States	Randomized controlled trial	Communication skills training for residents and NPs	Training significantly improved quality of communication with seriously ill patients in both resident physicians and nurse practitioners
31	CFM Resolution (48)	Brazil	Legal/regulatory document	Advance directives legal framework (Brazil)	Established formal Brazilian legal basis for advance directives (*diretivas antecipadas de vontade*); empowered patients to express end-of-life preferences in advance; complemented CFM Resolution 1,805/2006
32	Dadalto et al. (49)	Brazil	Legal/bioethical analysis	Brazilian advance directive model	Proposed standardized advance directive model adapted to the Brazilian legal and cultural context; identified implementation gaps in the healthcare system
33	Quill and Abernethy (61)	United States	Perspective	Generalist plus specialist palliative care model	Proposed sustainable model combining generalist and specialist palliative care; emphasized scalability and integration across care settings without dependence on specialist availability
34	Schneiderman et al. (60)	United States	Ethical analysis	Medical futility — meaning and ethical implications	Defined quantitative and qualitative futility; argued clinicians are not obligated to provide futile treatments; laid foundational conceptual framework for the futility doctrine
35	Lynch et al. (55)	International	Global mapping study	Development of palliative care capacity worldwide	Documented global disparities in palliative care development; identified regions with critical deficits in access and specialist training capacity
36	Dy et al. (68)	United States	Systematic review	Satisfaction with end-of-life care	Identified consistent determinants of patient/family satisfaction: communication quality, pain control, and emotional support; highlighted measurement challenges in end-of-life research

### Thematic distribution of included studies

The corpus of included studies was organized around eight thematic areas: (1) core clinical components of palliative care (symptom management, communication, decision-making); (2) ethical frameworks guiding ICU end-of-life decisions; (3) spiritual and existential care; (4) compassion, burnout, and clinician well-being; (5) family-centered care and surrogate decision-making; (6) barriers and facilitators to palliative care in Brazilian and Latin American ICUs; (7) legal and regulatory context in Brazil; and (8) comparative international perspectives on end-of-life practices. The majority of Brazilian studies employed observational or survey designs, while international contributions included systematic reviews, ethical analyses, and consensus statements. This methodological diversity is consistent with the integrative scope of the narrative review methodology employed.

## Discussion

### Fundamental aspects of palliative care in the ICU

The evidence synthesized in this review establishes that effective palliative care in the ICU is not reducible to the management of dying patients; rather, it constitutes a parallel layer of care that should run alongside curative and life-sustaining interventions throughout the trajectory of critical illness. Palliative care in the ICU integrates symptom relief, structured communication, shared decision-making, and family support offered by interdisciplinary teams. Evidence supports early integration of palliative care principles, standardized symptom assessment, structured family meetings, and team communication training to improve patient-and family-centered outcomes (5, 6). Decisions to withhold (not start) or withdraw (stop) life-sustaining treatments are ethically appropriate when the treatments no longer meet the patient’s goals or when the burdens outweigh the benefits (21, 22).

### Communication and goals of care

Structured communication is not merely a procedural component of palliative care in the ICU — it is the mechanism through which all other palliative interventions are coordinated and their ethical legitimacy established. Effective communication about prognosis, treatment options, and care goals is central to palliative care in the ICU. Regular and honest discussions with patients (when possible) and surrogate decision-makers help align care with patient values and preferences (6, 23). Systematic reviews and clinical guidelines endorse structured family meetings designed to improve understanding, reduce uncertainty, and support decision-making; they are associated with improved family coping, clearer care goals, and greater satisfaction with care (6, 23).

Clinical guidelines recommend communication skills training for ICU staff to increase the effectiveness of conversations about care goals. Training programs improve family satisfaction, the quality of decisions, and clinicians’ confidence in conducting difficult conversations (6, 23). In the Brazilian context, however, the structural barriers to implementing such communication practices are particularly pronounced: the absence of standardized family conference protocols, insufficient training in prognostic communication, and hierarchical team structures that limit the involvement of nursing and allied health professionals in goals-of-care discussions all compound the challenges identified in the international literature (8–10). The reviewed evidence suggests that communication training ideally covers skills such as conveying bad news, responding to emotions, eliciting values, and managing conflict — competencies that embody the virtues of truthfulness, empathy, and prudence described in the section on ethical frameworks below.

### Symptom management

The systematic assessment and management of physical suffering is the most immediate imperative of palliative care in the ICU. Unrelieved pain, dyspnea, and delirium constitute a form of iatrogenic harm inflicted on patients who are already among the most vulnerable in the healthcare system, and deficiencies in this domain carry direct ethical consequences. Effective symptom management in the ICU requires a structured, multimodal approach that integrates validated assessment tools, pharmacological and non-pharmacological interventions, and clinical reasoning anchored in individualized goals of care (5, 6, 24).

### Assessment as the foundation of symptom management

Systematic symptom assessment using validated instruments is the prerequisite for consistent and reproducible management. In non-communicative patients — the majority in mechanically ventilated ICUs — behavioral pain tools such as the Critical Care Pain Observation Tool (CPOT) and the Behavioral Pain Scale (BPS) are validated for pain assessment. Sedation-agitation is graded with the Richmond Agitation-Sedation Scale (RASS), which should guide analgesia-first protocols prioritizing comfort before sedation. Delirium is screened using the Confusion Assessment Method for the ICU (CAM-ICU); early identification allows timely non-pharmacological interventions. Standardized symptom screening at defined intervals — rather than *ad hoc* clinical judgment alone — is associated with earlier detection, more consistent symptom control, and fewer unnecessary interventions (6, 24).

### Pain

Pain is prevalent in ICU patients and remains inadequately controlled in a significant proportion despite advances in monitoring. Evidence-based management follows an analgesia-first approach: opioids (morphine, fentanyl, hydromorphone) titrated to effect remain the cornerstone for moderate to severe pain; multimodal analgesia combining opioids with adjuvants (acetaminophen, low-dose ketamine, gabapentinoids) reduces opioid burden and associated adverse effects (24). Crucially, opioid dosing should be guided by pain scores and patient response rather than predetermined dose ceilings. Non-pharmacological strategies — positioning, massage, reduction of noxious stimuli, and the therapeutic presence of familiar persons — address dimensions of pain and distress that pharmacological interventions alone cannot resolve and should be incorporated as standard components of the pain management plan (25).

### Dyspnea

Dyspnea is among the most distressing symptoms experienced by critically ill patients and is particularly prevalent in respiratory failure, heart failure, and malignancy. Systemic opioids are the most effective pharmacological intervention for alleviating respiratory distress and should not be withheld out of concern for respiratory depression when comfort is the explicit care goal — the principle of double effect supports their use when intention is symptom relief and doses are proportionate to suffering (21, 26, 27). Anxiolytics address the dyspnea-anxiety cycle. Non-pharmacological measures — positioning with head elevation, fan-directed airflow to the face, reduction of unnecessary stimuli, and reassuring presence — are effective adjuncts, particularly for awake patients. In mechanically ventilated patients, synchrony between ventilator settings and the patient’s respiratory effort is a clinical priority for comfort.

### Delirium

Delirium occurs in up to 80% of mechanically ventilated patients and is associated with increased mortality, prolonged ICU stay, and long-term cognitive impairment. It is a multifactorial syndrome requiring structured prevention and management rather than reactive pharmacological treatment. Non-pharmacological interventions constitute the first-line approach: structured reorientation, sleep–wake cycle restoration, early mobility, reduction of immobilizing devices, hearing and vision support, and minimization of sedative and anticholinergic medications — particularly benzodiazepines, which are independently associated with delirium (24). Pharmacological treatment with antipsychotics (haloperidol, quetiapine) may be appropriate for severe perceptual disturbances or agitation that compromises safety, though evidence for routine prophylaxis is limited. Terminal delirium — agitated delirium in the actively dying patient — is a distinct clinical presentation that may warrant proportional palliative sedation when non-pharmacological and standard pharmacological interventions fail to control refractory suffering (27).

### Nausea, secretions, and other distressing symptoms

Nausea and vomiting are underrecognized in ICU palliative care yet substantially impair patient comfort. Antiemetic selection should be guided by likely etiology: prokinetics for gastroparesis, antidopaminergic agents (haloperidol, metoclopramide) for opioid-induced nausea, and 5-HT3 antagonists (ondansetron) for broader coverage. Terminal secretions — pooling of pharyngeal secretions producing audible respiratory noise in unconscious patients — cause distress primarily to families and caregivers; repositioning and avoidance of unnecessary suctioning are first-line measures, with anticholinergic agents (glycopyrrolate, scopolamine) reserved for persistent cases. Opioid-induced constipation requires prophylactic stimulant laxatives initiated when opioids are started, not reactively. Pruritus and urinary retention related to opioids or other medications should be systematically assessed and treated. The systematic management of each of these symptoms reflects a commitment to comprehensive comfort that goes beyond pain and dyspnea alone.

### Non-pharmacological approaches and the broader concept of comfort

Non-pharmacological interventions are not secondary supplements to pharmacological care — they address dimensions of suffering that medications cannot reach: anxiety, loneliness, existential distress, and the loss of dignity. Evidence-based interventions include therapeutic music (25), structured family presence, therapeutic touch, and spiritual care interventions. Environmental modifications — controlling lighting, reducing noise, maintaining day-night cycles, and personalizing the patient’s space — are both fundamental to delirium prevention and independently improve patient and family comfort. The presence of a familiar person and structured, empathic communication between patients (where possible), families, and the clinical team are themselves therapeutic interventions for anxiety and distress, and should be treated as components of the symptom management plan rather than supplementary niceties.

### Ethical dimension of symptom management

The ethical principle of double effect supports the use of adequate doses of opioids and sedatives for symptom relief even when physiological effects could theoretically shorten life, provided the intent is symptom relief and doses are proportionate to suffering (21, 27). This principle is foundational to the ethical justification for palliative symptom management in the ICU and should be explicitly addressed in team communication, family discussions, and clinical documentation. Conversely, inadequate symptom control driven by unfounded concerns about double effect constitutes a significant ethical failure — one that compounds the suffering of already vulnerable patients.

### Terminal Extubation and palliative sedation

When mechanical ventilation is withdrawn, early management of dyspnea and distress is essential. Protocols for terminal extubation include premedication with opioids and sedatives, titration for comfort, and family preparation. Palliative sedation — proportional sedation to relieve refractory suffering — may be appropriate in selected cases, with careful attention to intention (relief of suffering, not hastening death) and ethical safeguards (26, 27).

### Ethical equivalence of withholding and withdrawing treatment

The distinction that clinicians and families often draw between not starting and stopping a life-sustaining treatment reflects psychological and cultural dynamics rather than ethically defensible differences — a point with direct consequences for Brazilian ICU practice, where the asymmetry between withholding and withdrawing remains a significant driver of disproportionate care. Most bioethicists and legal scholars internationally argue that there is no ethical or legal difference between withholding and withdrawing treatment, as the underlying intent — to avoid disproportionate burden — is the same. Both actions respect the patient’s autonomy and the principle of proportionality (21, 28). However, psychological and cultural factors often make withdrawal more difficult for clinicians and families, even when ethically equivalent (29).

### Artificial nutrition and hydration

Decisions about artificial nutrition and hydration (ANH) at the end of life are ethically complex and culturally sensitive. From a bioethical standpoint, ANH is considered a medical treatment that can be suspended or withdrawn when it no longer meets the patient’s goals or when the burdens outweigh the benefits (30, 31). The proportionality of ANH should be assessed against the benefits (e.g., prolonging life, improving comfort) versus the burdens (e.g., risk of aspiration, fluid overload, discomfort caused by probes). Decisions should reflect the patient’s previous values and desires when known. Food and water carry deep symbolic and emotional meaning for families, often representing care and love. Clinicians should address these meanings sensitively in discussions (32).

The claim that abstention from nutrition and hydration causes suffering through hunger and thirst in dying patients is not well supported by evidence. Studies in palliative care settings suggest that in the process of dying, patients typically do not feel hungry, and thirst can be controlled with oral care and small amounts of fluid (33, 34). However, this evidence comes primarily from palliative care settings, and extrapolation to all ICU settings should be done with caution. Providers should assess individual patient comfort and provide symptom control (oral care, cooling, nausea medications), rather than assuming universal experiences. Family-centered care recognizes that the family is the unit of care in the ICU; structured family meetings, psychological support, and postmortem bereavement programs improve family experience, coping, and long-term outcomes (6, 23).

### Ethical frameworks: complementing Principalism with virtue ethics

While principalism provides the foundational rules for medical ethics (autonomy, beneficence, non-maleficence, justice), virtue ethics focuses on the professional’s character and clinical reasoning. The literature suggests that virtue ethics complements principalism by providing a relational framework for addressing complex bedside dilemmas (13, 14).

### The role of virtue-based competencies

Rather than merely following rules, the virtuous clinician cultivates dispositions such as practical wisdom (*phronesis*), courage, and compassion. These are increasingly viewed as core professional competencies that guide clinicians in navigating conflicts between principles—for example, when a family’s request for “doing everything” conflicts with the duty to avoid non-beneficent treatments (13, 35).

The practical application of virtue ethics in the ICU includes: Practical Wisdom: Utilizing clinical experience and moral insight to individualize care levels when guidelines are ambiguous, Relational Attentiveness: Prioritizing the quality of interaction and empathy during goals-of-care discussions, which has been linked to higher levels of trust and reduced family-perceived conflict (13, 36), and Mitigating Moral Distress: Teams focused on shared values and the cultivation of character-based resilience report lower rates of burnout and moral distress (11, 12, 37).

### Compassion in end-of-life care

Compassion — defined as sensitivity to suffering coupled with a commitment to alleviate it — is not an optional supplement to technical competence in the ICU; it is the motivational foundation that distinguishes palliative care from mere symptom management. Compassion is central to palliative care and is increasingly recognized as a cultivable skill rather than an innate trait (38, 39). Healthcare professionals’ understanding of compassion includes relational, cognitive, and behavioral dimensions: acknowledging suffering, connecting emotionally with the patient, and taking action to alleviate suffering (39). Compassion differs from empathy (feeling with the other) in that it includes the motivational component to act.

Contrary to previous assumptions, current evidence supports that compassion can be developed through structured, experiential, and reflective training programs. Multi-intervention studies demonstrate measurable increases in self-reported competence in compassion, self-compassion, and trust in compassion after training among practicing clinicians (40–42). Neurobiological accounts indicate that neural circuits related to compassion can be shaped through practice (neuroplasticity), supporting the plausibility of adult learning of compassion skills (43). Meta-analyses and systematic reviews conclude that compassion-based interventions can reduce compassion fatigue and increase compassion and self-compassion among health care providers, although the heterogeneity of studies is substantial and data on objective long-term outcomes remain limited (40, 44).

Cultivating compassion and self-compassion can reduce moral distress and burnout among ICU professionals. Contemplative end-of-life training programs report from participants reduced moral distress, greater ability to be present, and better coping with grief and burnout (37). Self-care programs in palliative care teams improved emotional regulation, reduced fatigue, and expanded team communication — relevant mechanisms to reduce moral distress in critical care settings (45, 46).

### Spiritual care in the ICU

Spiritual care addresses the existential, meaning-making, and transcendent dimensions of human experience. In the ICU, spiritual distress is common among patients and families facing critical illness and death, and meeting spiritual needs is an integral part of comprehensive palliative care (15, 16).

Spiritual distress in ICU palliative care describes the non-physical existential distress that patients and families experience when facing critical illness and death. Commonly, it involves loss of meaning, identity fracture, moral or existential distress, and disrupted relationships or rituals (15, 47). Spiritual and existential suffering is a prominent feature of dying and can be expressed along the trajectory before, during, and after death, shaping the experience of patients, families, and clinicians (15). Nursing and qualitative studies characterize non-physical distress as “identity fracture” with existential and moral dimensions that require discernment beyond physical symptom control (47).

### The Brazilian context: challenges and cultural factors

Understanding why Brazilian ICUs consistently underperform relative to international standards in the provision of palliative care requires more than a catalog of individual barriers — it requires situating those barriers within the specific legal, cultural, and systemic context that has shaped Brazilian end-of-life practice over the past two decades. The Brazilian reality is characterized by a persistent and well-documented gap between bioethical consensus, which supports the ethical appropriateness of life support limitation, and clinical practice, which continues to be driven by legal fears, insufficient training, and cultural values that equate withdrawal with abandonment (8–10).

#### Specific obstacles in Brazilian ICUs

Brazilian ICUs report multiple practical and systemic barriers that limit the provision of palliative care. Legal concerns, insufficient training, and fragmented staffing processes reduce the frequency of active limitation of life support and hinder the adoption of palliative measures (8–10).

#### Legal uncertainty

Legal uncertainty has been repeatedly identified as one of the main barriers to limiting therapeutic effort in Brazilian ICUs (8, 9). Despite the resolutions of the Federal Council of Medicine of Brazil (CFM) that clarify the ethical adequacy of orthothanasia (allowing natural death without futile interventions), many professionals fear legal repercussions for withholding or withdrawing life support. This fear persists even in the absence of legal precedent for the prosecution of clinicians acting in good faith to honor patients’ wishes and avoid futile treatments (48, 49).

CFM Resolution 1,805/2006 explicitly allows physicians to limit or suspend treatments that prolong death when there is no therapeutic benefit, as long as the decision respects the patient’s autonomy and is documented (50). This resolution was subsequently upheld by CFM Resolution 1,995/2012 on advance directives, which created the formal legal framework for patients to express their end-of-life preferences in advance (48). However, awareness and implementation of these resolutions remain inconsistent in Brazilian ICUs.

#### Inadequate training

Lack of education about end-of-life care is common among Brazilian ICU professionals. Only a minority report adequate training, and educational gaps are associated with more aggressive practices and less use of DNR orders (9, 10). Palliative care education is not uniformly integrated into medical, nursing, and health curricula in Brazil, and opportunities for continuing education in palliative care in the ICU are limited (51, 52).

#### Team fragmentation and specialist resistance

Problems of team integration and resistance from specialists hinder the implementation of palliative care plans, especially in oncology and pediatric settings (10, 2). Multidisciplinary collaboration is often hampered by hierarchical structures, poor communication, and a lack of shared decision-making protocols. Specialists may resist palliative transitions, seeing them as “giving up” rather than appropriate changes in care goals (2).

#### Gaps in communication and symptom management

Deficiencies in communication skills and symptom management overwhelm multidisciplinary practice and contribute to moral distress and burnout among staff (11, 53). Structured family meetings are not routinely held, and clinicians often lack training to convey bad news, elicit values, and manage conflict (54).

#### Preference for withholding over withdrawal

A generalized preference for withholding rather than withdrawing treatment is observed in Brazilian ICUs. Clinicians and staff are more comfortable with not escalating care than actively removing life support, even when continuing support prolongs distress (9, 10).

#### Fear of social and institutional repercussions

Fear of social or institutional repercussions and a cultural emphasis on “doing everything possible” can delay transitions to comfort-focused care and shape prognostic discussions (2, 8). Practitioners may avoid frank prognostic discussions or recommendations to limit treatment due to concerns about family reactions, institutional criticism, or legal consequences (9).

### Strategies to individualize life support levels and prevent dysthanasia

While identifying barriers to palliative care represents a necessary analytical step, the ultimate goal of this review — and the clinical imperative for Brazilian ICUs — is to translate the evidence base into actionable, context-sensitive strategies that individualize life support decisions and prevent dysthanasia. The following evidence-based recommendations integrate international guidance with the specific regulatory and cultural tools available in the Brazilian context.

1. *Individualization of life support based on the proportionality principle*: The principle of proportionality — assessing the benefit-to-burden ratio of each intervention in light of the patient’s prognosis and goals — should serve as the foundation for all life support decisions. In practice, this requires the use of validated prognostic scoring tools (e.g., the Sequential Organ Failure Assessment [SOFA] score) to communicate clinical trajectories objectively, alongside explicit discussions of qualitative futility when physiological targets are achievable, but holistic patient benefit is absent (55). Clinical teams should adopt explicit criteria for identifying patients who may benefit from palliative care consultation, including clinical indicators of high mortality risk, prolonged ICU stay, and documented poor prognosis.
2. *Early and structured advance care planning*: Advance care planning — including structured goals-of-care discussions, formal documentation of patient preferences, and the use of advance directives (*diretivas antecipadas de vontade*) is the most effective tool for aligning life support decisions with individual patient values and preventing dysthanasia prospectively. CFM Resolution 1,995/2012 (48) provides the legal framework for advance directives in Brazil; however, their implementation in ICUs remains limited (49). Healthcare teams should proactively offer advance care planning conversations to patients with life-threatening diagnoses at the time of ICU admission, rather than reserving them for the terminal phase.
3. *Structured goals-of-care conferences and DNR documentation*: Standardized protocols for family conferences, including scripted frameworks for eliciting patient values and communicating prognosis, improve the quality of end-of-life decisions and reduce family distress (6, 23). Early documentation of DNR/DNAR orders, based on prospective goals-of-care discussions, prevents disproportionate resuscitation attempts at the end of life. Institutions should adopt trigger-based systems for mandatory palliative care consultation (e.g., ICU admission with metastatic malignancy, multiple organ dysfunction > 7 days, or documented patient refusal of life-sustaining treatment).
4. *Institutional ethics committees as decision-support structures*: The role of institutional ethics committees in supporting clinicians who face pressure to continue futile treatments has been insufficiently explored in the Brazilian context. Ethics committees can provide a structured forum for case review, promote adherence to CFM resolutions (50, 48), and serve as an institutional buffer against the perceived legal risk that drives dysthanasia. Their expansion and activation in Brazilian ICUs represent a concrete strategy to bridge the gap between bioethical consensus and clinical practice.
5. *Engagement with the Brazilian palliative care academy consensus*: The 2024 position statement of the Brazilian Palliative Care Academy (*Academia Nacional de Cuidados Paliativos*, ANCP) — authored by Vidal et al. and published in *Critical Care Science* — addresses specifically the criteria for withholding and withdrawing life-sustaining interventions in the context of palliative care in Brazil (56). This landmark document represents the most authoritative and current national expert consensus on the topic and should serve as a direct reference for institutional protocols, educational curricula, and clinical guidelines in Brazilian ICUs.

### Comparison with international literature

The comparison of Brazilian ICU practice with international data reveals not merely a quantitative gap in life support limitation rates, but a qualitatively distinct ethical and institutional landscape shaped by specific legal, cultural, and historical factors. Brazil consistently shows lower rates of active life support limitation, greater preference for withholding over withdrawal, and distinct legal and cultural determinants of practice compared to North American and European settings (9, 10, 57). Historically, Brazilian and Latin American ICUs have reported lower rates of life support limitation than North America and Europe (57). Although pediatric life support limitation rates in Brazil have increased over time, they remain lower than in many high-income countries (9).

*Withholding preferred over withdrawal*: Withholding is more favorable relative to withdrawal in Brazil than in many international series where withdrawal is the more commonly practiced mode of end-of-life decision (9, 10). International data show that mechanical ventilation withdrawal is a common mode of death in ICUs in the United States, Canada, and parts of Europe, while Brazilian ICUs more often decline to escalate while maintaining existing support (57).

- *Contrasts in family involvement*: Brazilian studies note lower family participation in decisions, although families are often in favor of non-aggressive care (9, 58). This contrasts with North American models where shared decision-making with active family involvement is more established (59).
- *Latin American comparative context*: Brazil’s challenges in implementing end-of-life palliative care are shared, in varying degrees, with other Latin American countries, and the comparative analysis is instructive for identifying both common obstacles and potential solutions. Colombia enacted Law 1733/2014 (*Ley Consuelo Devis Saavedra*), which legally recognized the right to palliative care and formalized the limitation of therapeutic effort as a patient right, providing a more explicit legal foundation than currently exists in Brazil beyond CFM resolutions. Argentina has made moderate advances in national palliative care coverage, as documented in the WPCA global atlas; its National Palliative Care Program, though underfunded, established institutional frameworks for end-of-life care in the public health system. Mexico incorporated palliative care provisions into the General Health Law in 2009, creating a federal mandate for access to palliative care, although implementation has been uneven across states and healthcare systems. These countries share with Brazil the cultural, structural, and historical challenges of implementing end-of-life care in predominantly Catholic societies with resource-constrained public health systems — including the influence of religious values on death-related decisions, limited specialist training, and the relative novelty of palliative care as a formal medical specialty. However, the existence of explicit legislative frameworks in Colombia and Mexico suggests that legal reform may be a prerequisite for overcoming the clinical culture of legal fear that drives dysthanasia in Brazilian ICUs.

The ETHICUS and ETHICUS-2 studies (Sprung et al.) provide robust comparative international data on end-of-life practices in European ICUs, documenting substantial north–south variation even within Europe in the rates and modes of treatment limitation (57). These findings suggest that cultural, legal, and institutional factors exert stronger influence on end-of-life practice than medical or technological variables alone — a conclusion with direct relevance for Brazil.

### Legal and regulatory context

The legal and regulatory context has uniquely shaped practice in Brazil, and understanding its trajectory is essential for appreciating both the progress achieved and the gap that remains. Past legal uncertainty restricted the adoption of life support limitation to clarifications issued by professional regulatory bodies (CFM resolutions), producing a distinct trajectory compared to countries where explicit legal frameworks for end-of-life care were already established (48, 49, 57).

### Ethical tensions in Brazilian practice

The persistence of ethical conflicts in Brazilian ICUs — around who decides, what constitutes futility, and whether limitation (including palliative extubation) is ethically and legally acceptable — reveals a healthcare system in which institutional and cultural inertia has not yet been overcome by regulatory clarity or educational reform (9–11).

#### Perceived legal risk vs. clinical judgment

Perceived legal risk leads practitioners to act against what they believe is best for patients, creating an ethical-practical gap (9). Despite the CFM resolutions clarifying the legality of orthothanasia, the fear of prosecution or institutional censorship persists (8, 48). This fear is not supported by legal precedent — there are no documented cases of Brazilian physicians being prosecuted for limiting life support in accordance with patients’ wishes and professional guidelines — but it remains a powerful barrier (49).

#### Ambiguity about futility and proportionality

Ambiguity about the futility and proportionality of interventions complicates consensual decision-making and can prolong non-beneficial treatments (11, 60). The concept of “futility” is contested: medical futility (treatments that do not achieve physiological goals) is relatively clear, but qualitative futility (treatments that achieve physiological goals but do not benefit the patient holistically) involves value judgments (60). Brazilian ICUs often lack explicit criteria or processes to determine futility, leading to inconsistent practices and prolonged aggressive treatments that increase suffering without meaningful benefit (10, 11).

#### The gap between bioethical consensus and clinical practice

A striking feature of the Brazilian context is the gap between the established bioethical consensus and actual clinical practice. International and Brazilian literature on bioethics, professional guidelines, and legal frameworks — including the 2024 position statement of the Brazilian Palliative Care Academy (56) — support the ethical appropriateness of withholding and withdrawing life-sustaining treatments when they no longer meet the patient’s goals (21, 28, 48). Yet clinical practice often does not follow this consensus (9, 10).

### Institutional and systems changes

Institutional change is the necessary substrate for translating ethical consensus into clinical practice. Individual clinician values and skills cannot overcome systemic barriers in the absence of organizational structures that support, incentivize, and protect palliative care decision-making. The following system-level interventions represent the highest-yield strategies for Brazilian ICUs:

- *Establish palliative care consultation services*: Develop specialized teams available for consultation in ICUs (55, 61).
- *Implement protocols and checklists*: Create standardized protocols for family conferences, symptom management, terminal extubation, and DNR orders (62, 63).
- *Promote interdisciplinary collaboration*: Foster team-based decision-making, with clear roles for physicians, nurses, social workers, and chaplains (59).
- *Integrate spiritual care*: Ensure routine spiritual screening, timely referral to chaplains, and chaplain participation in family meetings (64, 65).
- *Support staff well-being*: Offer debriefing sessions, peer support, mental health resources, and self-care programs to reduce moral distress and burnout (66, 67).
- *Conduct local research*: Generate Brazilian data on palliative care practices, barriers, and outcomes to inform context-specific interventions (9, 10).
- *Implement quality metrics*: Track indicators such as palliative care consultation rates, family satisfaction, symptom control, and DNR documentation (68).
- *Evaluate interventions rigorously*: Rigorously evaluate educational programs, protocols, and system changes to identify effective strategies (69).

### Synthesizing the evidence: toward a Brazilian model for ICU palliative care

The thematic synthesis presented in this review converges on a central finding: the fundamental aspects of ICU palliative care — symptom management, communication, ethical decision-making, family support, and spiritual care — are well established in the international evidence base, but their translation into Brazilian ICU practice is impeded by a cluster of mutually reinforcing barriers that operate simultaneously at the individual, team, institutional, and regulatory levels. Legal uncertainty amplifies cultural reluctance to withdraw treatment; inadequate education perpetuates the ethical-practical gap; team fragmentation undermines the interdisciplinary model on which palliative care depends; and the absence of institutional infrastructure leaves individual clinicians without the organizational support they need to act on their ethical convictions.

What distinguishes the Brazilian scenario from other resource-constrained settings is not the absence of ethical consensus — the Brazilian Palliative Care Academy, the CFM, and the broader bioethics community have produced clear guidance — but the failure to operationalize that consensus at the bedside. Addressing this gap requires simultaneous action on multiple fronts: legal clarification and dissemination of the existing CFM framework; integration of palliative care and end-of-life communication into undergraduate and postgraduate health curricula; development of institutional protocols anchored in the proportionality principle and the recommendations of Vidal et al. (56); and the cultivation of a professional culture in which compassion, practical wisdom, and attention to dying as a human experience are understood as core ICU competencies rather than optional additions. The insights drawn from this national reality — and the resonance with comparable challenges documented in Colombia, Argentina, Mexico, and other Latin American countries — suggest that the strategies proposed here have relevance beyond Brazil for health systems facing similar end-of-life dilemmas.

## Conclusion

Palliative care is an integral part of the ICU, encompassing symptom management, structured communication, family and spiritual support, and ethical decisions about withholding or withdrawing treatments. In the Brazilian scenario, implementation faces critical barriers, including legal uncertainty, inadequate training, cultural reluctance, and fragmentation of teams. These obstacles generate ethical tensions and moral distress, distancing clinical practice from bioethical consensus. Virtue ethics and spiritual care emerge as essential complements to principalism, offering practical wisdom and relief from existential suffering in a context of strong religious influence.

Overcoming these challenges requires multifaceted strategies: legal clarification of the existing CFM framework; integration of palliative care into medical education; institutional protocols grounded in the proportionality principle; spiritual screening; early advance care planning; structured goals-of-care conferences; and support for the well-being of professionals. The 2024 position statement of the Brazilian Palliative Care Academy (56) provides an authoritative national consensus reference to guide this process. By adopting these measures, Brazilian ICUs can migrate to a more compassionate model that is aligned with the patient’s goals and that prevents dysthanasia through institutional, educational, and cultural transformation.
